# Glances at the Hospitals

**Published:** 1901-05-11

**Authors:** 


### GLANCES AT THE HOSPITALS.

### THE EAST SUFFOLK AND IPSWICH HOSPITAL.

The committee bemoan the fact that there is a constantly increasing expenditure, and that this has not been accompanied by a corresponding increase in the annual income. Indeed, the expenditure for the year under notice exceeded the income by £899; and the committee believe that this deficit will be increased next year by the opening of the new wing. It seems certain that another £1,000 must be forthcoming or the committee must economise in one direction or another. The total expenditure of the year was £4,827, and the total receipts £3,927. The subscriptions amounted to £1,660, and, although this hospital has a considerable sum invested, it is chiefly on its regular subscriptions that it must look for the bulk of its income. It does not appear what proportion of this £1,660 was subscribed by the workpeople themselves; nor do the rules state what, if any, limit is placed on the wages earned by a patient above which limit charitable medical treatment will be refused. At the beginning of the year there were 78 patients in the hospital, and 805 were admitted, making a total of 883 under treatment. Of these 502 were discharged cured, 155 much benefited, 58 improved, 45 for other reasons, 52 died, and 71 remained in the hospital. The out-patients reached a total of 8,928, and of these 8,418 were cured, relieved, or made in-patients, and 510 remained under treatment. No medical or surgical tables of diseases are given; but a list of the operations performed is printed, and it shows the results. An extra column with remarks by the surgeon would be desirable, and also a table showing the kind of anæsthetic employed. It is stated that 140 ophthalmic operations were performed, but no results are given.

### THE SOUTHPORT INFIRMARY.

During the year an important step was taken in amalgamating the Infirmary with the Eye, Ear, and Throat Hospital. The amalgamation has been carried out "on terms which secure efficiency without that waste of effort inseparable from practically competing institutions." We believe this union to be a desirable step. The total ordinary income of the year was £3,252, and the total ordinary expenditure was £3,702. Last year there was an adverse balance of £587, and this year it has swollen to £1,281. It should, however, be noted that legacies to the amount of £9,207 have been received. The average weekly cost per head for diet was 6s. At the beginning of the year the hospital contained 53 patients, and 508 were admitted during 1899, making a total of 561. Of these 226 were medical cases, 260 surgical, 62 were accidents, and 13 gynæcological cases. Of the total 388 were discharged cured, 61 relieved, 15 for other reasons, 51 died, and 46 remained under treatment. A table of the medical and surgical cases is given, but it merely states the number of patients suffering from the various diseases; hence it is practically useless, although there is a cause of death table. The list of operations shows the results.

### THE ROYAL DEVON AND EXETER HOSPITAL.

This hospital has now the right to the term "Royal," which honour was conferred on it by the Queen. The total cash receipts for the year amounted to £13,698; but this includes the sum of £3,347 realised by the authority of the governors for credit of the building fund, and various legacies, benefactions, &c. The total expenditure amounted to £12,137, which includes the £3,347 passed to the building account, and a sum of £230 spent on the Operating Theatre and other work. There were 1,322 in-patients and 4,303 out-patients under treatment during the year. Of the in-patients 414 were discharged cured, 76 improved, 563 made out-patients, 15 as incurable, 48 for other reasons, 62 died and 145 remained in the hospital. The medical and surgical diseases are tabulated, but no results are given. Again, there were 490 operations performed during the year, but nothing is said as to the success or otherwise of any of them. It is to be hoped that these faults will be remedied in the next report, and that a new table will be inserted showing the nature of the anæsthetics employed.

